# Nutritional Screening Tools in the Pediatric Population: A Systematic Review

**DOI:** 10.3390/nu17030433

**Published:** 2025-01-24

**Authors:** Carlos Veiga Fachal, Sara María Fernández-González, Ana Moreno-Álvarez, Alfonso Solar-Boga

**Affiliations:** Pediatric Gastroenterology, Hepatology and Nutrition Unit, Department of Pediatrics, A Coruña University Hospital, Area Sanitaria A Coruña-Cee, 15006 A Coruña, Spain; carlosveif@gmail.com (C.V.F.); sara.maria.fernandez.gonzalez@sergas.es (S.M.F.-G.); alfonso.solar.boga@sergas.es (A.S.-B.)

**Keywords:** malnutrition, pediatric population, nutritional risk, nutritional screening tool, systematic review

## Abstract

Background/Objectives: Disease-related malnutrition (DRM) is the most common type of malnutrition in industrialized countries, and it has a high associated morbidity. Despite the existence of various screening tools for its detection, there is currently no consensus in the literature on which one is the most appropriate for clinical use. The aim of this systematic review is to update the available evidence on pediatric nutritional screening tools and to compare their validity and applicability. Methods: A systematic review of the literature was conducted using the MEDLINE (PubMed) database, selecting articles related to nutritional screening tools in the pediatric population. A quality assessment was carried out using the Quality Assessment of Diagnostic Accuracy Studies 2 (QUADAS-2). Results: A total of 200 articles were identified, of which 11 were included in the review. They include a total of 9,573 patients (mean age of 6.7 years and 49% female) from different countries. Eight screening tools were found, and three of them were the most employed: the Screening Tool for Risk on Nutritional Status and Growth (STRONGkids), the Screening Tool for the Assessment of Malnutrition in Pediatric (STAMP), and the Pediatric Yorkhill Malnutrition Score (PYMS). A high level of heterogeneity was observed among the selected studies without a gold standard for comparison. Conclusions: Despite the heterogeneity, the PYMS seems to have the greatest capacity to detect pediatric patients at nutritional risk and should therefore be considered when choosing a nutritional screening tool.

## 1. Introduction

According to the World Health Organization (WHO), malnutrition is a condition in which the body’s nutritional needs are not met due to insufficient intake, poor absorption, or inadequate utilization of nutrients [1,2].

There are different types of malnutrition depending on its cause: primary or exogenous malnutrition, resulting from a lack of access to food, and secondary or endogenous malnutrition, which occurs as a consequence of disease [3]. In our environment and in other industrialized countries, the most common cause of malnutrition is disease-related malnutrition (DRM), especially in patients who require hospitalization [4,5]. DRM differs from nosocomial malnutrition, as nosocomial malnutrition refers to the nutritional imbalance acquired during hospital stays [6].

The prevalence data available in the literature are highly heterogeneous as they depend on the criteria used to define malnutrition, as well as the standards and/or growth charts used as reference [7,8]. The estimated prevalence of DRM in the pediatric population ranges from 5 to 31% for acute malnutrition and from 12 to 18% for chronic malnutrition [7,9,10,11,12,13,14], and it can reach up to 44% in conditions with high nutritional risk, such as cardiac and renal diseases [7].

It is important to identify DRM early and accurately, as timely nutritional intervention can help reduce prolonged hospital stays, the risk of comorbidities (e.g., immunosuppression, muscle atrophy, and delayed wound healing), hospital readmissions, and mortality rates [3,4,6,14,15,16,17,18,19,20].

To facilitate nutritional screening, various tools have been developed to enable the early recognition of malnutrition [3,15], allowing for the implementation of early measures to prevent clinical deterioration. These tools vary in terms of the population they are designed for, the parameters they assess, and the reference standards they use [17,18,19,21,22,23,24,25,26,27,28]. An ideal screening tool would be one that is quick and easy to apply, reproducible, and capable of identifying individuals at higher risk who may benefit from a more in-depth evaluation later on [14]. Therefore, these nutritional screening tools do not replace a complete nutritional evaluation, which should be reserved for selected patients [4].

The literature contains numerous published articles aimed at studying the validity and applicability of these tools [9,10,11,15,20,27,29,30,31], comparing them, and determining which is best suited for routine clinical practice, but no definitive conclusions have been reached.

For all of these reasons, we conducted this systematic review with the aim of updating the available evidence on nutritional screening tools in pediatric patients, analyzing the main characteristics of the included studies, and comparing the validity of these scales based on the reference parameters used: anthropometric assessment, specialized nutritional evaluation, and/or long-term weight evolution.

## 2. Materials and Methods

This review was conducted in accordance with the PRISMA (Preferred Reporting Items for Systematic Reviews and Meta-Analyses) guidelines [32]. To structure the review process, the PICOS model (Population, Intervention, Comparison, Outcomes, and Study) was followed [33] (see Table 1).

Before starting the systematic review, the study was registered in the International Prospective Register of Systematic Reviews (PROSPERO) with ID No. CRD42024549443.

A systematic literature review was conducted to identify relevant publications on nutritional screening tools in the pediatric population. The search was carried out in the MEDLINE (PubMed) database, and published observational, cross-sectional, assessment, and validation studies were included. This review includes studies published between 1 November 2017—because the last systematic review included articles up to that date [27]—and 1 April 2024, which is the date of protocol and PROSPERO registration.

The descriptors were chosen according to the Medical Subject Headings (MeSH) list: “malnutrition,” “diagnostic screening programs,” and “diagnosis.” In addition to the descriptors, the Boolean operator “OR” was applied between “diagnosis” and “diagnostic screening programs,” and the Boolean operator “AND” was used between “malnutrition” and the other two for term combination in the databases. Additionally, the filters “Observational Study,” “Comparative Study,” “Evaluation Study,” “Validation Study,” “Humans,” and “Child: from birth to 18 years” were applied. No language restrictions were imposed.

Two authors (S.M.F.G. and C.V.F.) independently read the titles and abstracts of all identified articles. In case of discrepancies, a third evaluator (A.M.A.) was consulted. Selected articles in full text were obtained from potentially eligible studies for further evaluation. The four reviewing authors (S.M.F.G., C.V.F., A.M.A., and A.S.B.) agreed on the final list of included studies.

Two reviewers (S.M.F.G. and C.V.F.) independently extracted the data. The following information was collected: author name, year of publication, study design, applied screening tool, ages and pathology of the selected patients, results of the screening tools, results of the reference standard used, and information necessary to assess the risk of bias.

Two researchers (S.M.F.G. and C.V.F.) independently assessed the risk of bias of the included studies using QUADAS-2 (Quality Assessment of Diagnostic Accuracy Studies 2) [34]. In case of disagreement, a third author (A.M.A.) resolved the discrepancy.

The data obtained from the included studies were synthesized narratively. A meta-analysis was not conducted because the reporting of results (lack of numerical results) was incomplete, the effect measures used in the included studies varied, and there were relevant methodological and clinical differences among the studies.

Quantitative variables were expressed as means, preferably, or medians, depending on availability. Qualitative variables were expressed as percentages.

This systematic revision compliance with the PRISMA guidelines and elements of the PRISMA checklist [32] are included in Supplementary Material (Supplementary Tables S1 and S2).

## 3. Results

**Figure 1:**
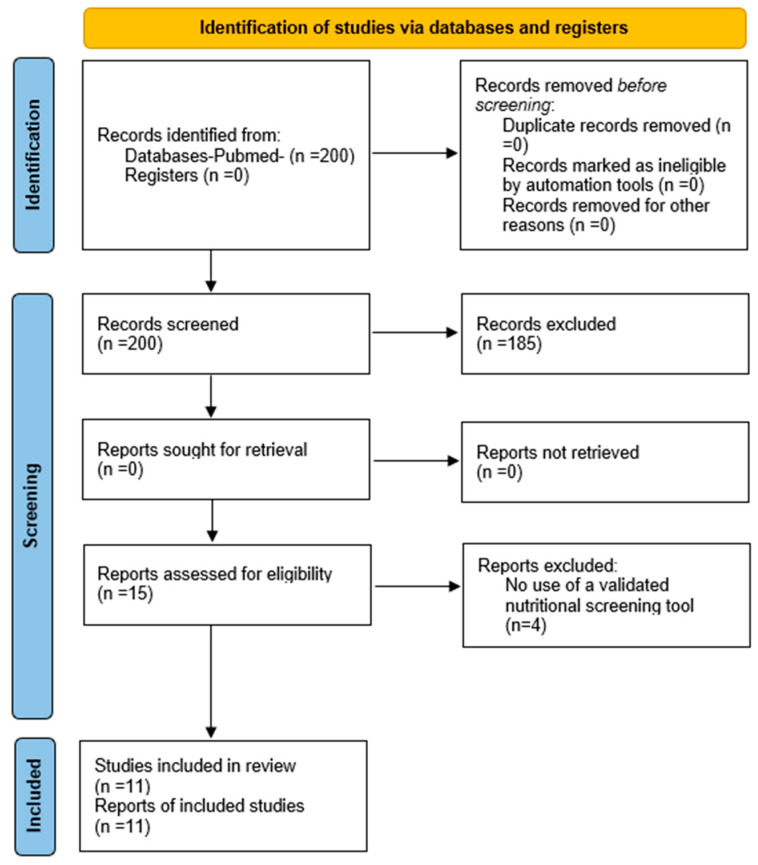
Flowchart of article selection for systematic review, according to PRISMA (Preferred Reporting Items for Systematic reviews and Meta-Analyses) model 2020 [32].

A total of 200 articles were obtained from the initial search. Titles and abstracts were screened, resulting in the exclusion of 165 articles. A full-text review of the remaining 35 articles was conducted, of which 15 were selected for full evaluation based on the inclusion and exclusion criteria. Finally, 11 articles were included in the review [28,35,36,37,38,39,40,41,42,43,44]. This selection is summarized in the flowchart (Figure 1), and the primary reasons for exclusion of the remaining studies can be found in the Supplementary Material (Supplementary Table S3).

The selected articles were published between 2018 and 2022, with four of them being published in 2018 [28,37,38,39]. Six studies were conducted in Europe [36,38,40,41,42,44], four in Asia [28,37,39,43], and one in South America [35].

Most of the studies included the pediatric population with different pathologies, although one study exclusively included patients attending emergency services [35], two studies focused on oncology patients [40,42], and one study on burn patients [39].

In total, the articles included 9573 patients (49% female) with a mean age of 6.7 years. Five articles (45.5%) excluded neonatal populations [28,35,37,38,41].

Regarding the reference standards used, six articles employed anthropometry [35,37,38,39,41,42], one used evaluation by a nutritionist [43], three combined anthropometric assessment and nutritionist evaluation [27,37,45], and one compared the Screening Tool for Childhood Cancer (SCAN), the Nutrition Risk Screening for Pediatric Cancer (NRS-PC), and bioimpedance analysis [40].

Only one of the studies had a low risk of bias [43] according to the QUADAS-2 classification. The rest either did not use an appropriate reference standard (employing anthropometry or other screening tools) [35,37,38,39,40,41,42], or the evaluation by a nutritionist was not applied to the entire sample [28,36,44]. Furthermore, in many of these studies [35,37,40,41,42], it was not specified whether different researchers evaluated the diagnostic test and the reference standard.

None of the studies reported the time intervals between the index test and the reference test.

The results of the methodological quality according to QUADAS-2 [34] are presented in Table 2.

A total of eight screening tools were employed in the studies, including the Screening Tool for Risk on Nutritional Status and Growth (STRONGkids), the Screening Tool for the Assessment of Malnutrition in Pediatric (STAMP), the Pediatric Yorkhill Malnutrition Score (PYMS), the Pediatric Nutritional Risk Score (PNRS), SCAN, NRS-PC, the Pediatric Nutritional Screening Score (PNSS), and Handgrip Strength (HGS). The most commonly used tools were STRONGkids, which was used in five articles [28,35,37,39,41], as well as PYMS [28,36,37,39,44] and STAMP [28,36,39,41,43]. SCAN was used in studies conducted on oncology patients [41,43]. In one study, NRS-PC was also included, along with a bioelectrical impedance analysis, as previously mentioned [40]. Additionally, the PNRS tool was used within the Evalnut software [38].

Finally, two other screening tools were used, PNSS [28] and HGS, which were proposed as screening tools at the pediatric level [45].

**Figure 2:**
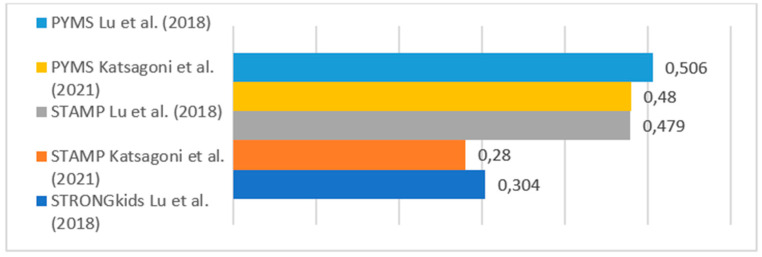
Kappa values of PYMS, STAMP, and STRONGkids. Lu et al. (2018) [28], Katsagoni et al. (2021) [36].

**Figure 3:**
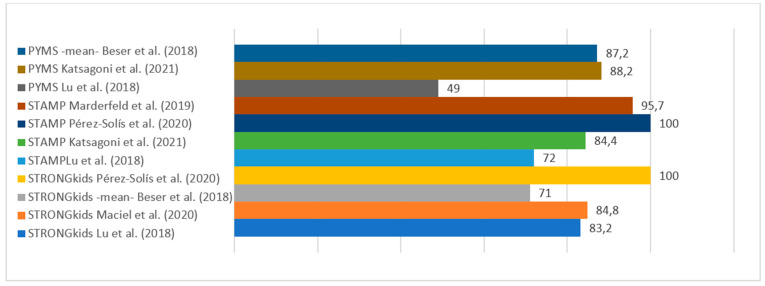
Sensitivity values of PYMS, STAMP, and STRONGkids. Beser et al. (2018) [37], Katsagoni et al. (2021) [36], Lu et al. (2018) [28], Marderfeld et al. (2019) [43], Pérez-Solís et al. (2020) [41], Maciel et al. (2020) [35].

**Figure 4:**
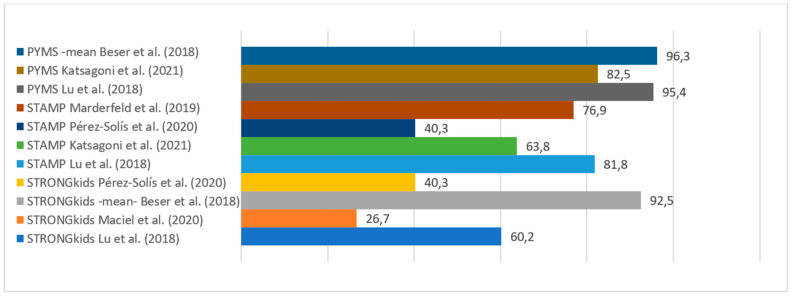
Specificity values of PYMS, STAMP, and STRONGkids. Beser et al. (2018) [37], Katsagoni et al. (2021) [36], Lu et al. (2018) [28], Marderfeld et al. (2019) [43], Pérez-Solís et al. (2020) [41], Maciel et al. (2020) [35].

Table 3 and Figure 2, Figure 3 and Figure 4 shows the descriptive summary of the analyzed studies.

They also collected the variables of sensitivity, specificity, positive predictive value, and negative predictive value, as well as the kappa between tools and the Area Under the Curve (AUC) in the articles that provided these data. These results are summarized in Table 4. As mentioned before, the heterogeneity in the included studies and the risk of bias does not allow for an adequate comparison in a meta-analysis. 

As previously mentioned, a screening tool should be simple and able to identify individuals at higher risk so that they can be referred early for further evaluation (which would correspond to a test with high sensitivity). However, this increased sensitivity is linked to a loss of specificity after a certain point, as we can see in the study by Pérez-Solís et al. [41], where STAMP and STRONGkids have high sensitivity (100%) at the cost of low specificity (40 and 48%, respectively), and therefore would not be efficient as screening tools.

Finally, regarding hospital stay duration, we noted that the high nutritional risk group was significantly associated with a longer hospital stay, as evaluated by STRONGkids, PYMS, and STAMP (*p* < 0.05) [37,39] as well as by PNRSS [38] and PNSS [28].

## 4. Discussion

DRM constitutes a significant problem in the pediatric population due to its high prevalence and associated morbidity and mortality [3,4,6,7,9,10,11,12,13,14,15,16,17,18,19,20]. Detecting these patients enables early intervention, aiming to stop and reverse their nutritional deterioration.

In our review, we analyzed the available evidence on nutritional screening tools used in the pediatric population. Although eight tools were identified, three of them (STRONGkids, STAMP, and PYMS) were the most used, suggesting a trend toward their preference in clinical practice, which aligns with previous observations in the literature [28,35,36,37,38,39,40,41,42,43,44]. However, most of the studies included in our review presented a high risk of bias, which limits the robustness of the conclusions and highlights the need for further research with higher methodological quality.

STRONGkids was used in five [28,35,37,39,41] of the eleven articles included in our review. It is characterized by a subjective examination of subcutaneous fat, pathology, nutritional intake and losses, and weight loss reported in the period prior to admission [17]. Maciel et al. [35] compared anthropometry with this scale, concluding that it allows for the early identification of nutritional risk due to its high sensitivity (84.8%), finding a low correlation between the nutritional screening tool and anthropometry. Otherwise, other studies have shown a statistically significant correlation between anthropometry and the STRONGkids assessment (*p* < 0.001) [20].

**Figure 5:**
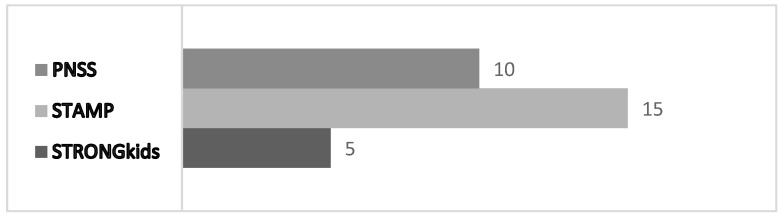
Average time needed for screening [28,31].

The absence of anthropometry in some tools increases their applicability, simplifies their use, and reduces the time required for evaluation [25]: 5 min is needed for STRONGkids compared to 15 min for STAMP and 10 min for PNSS [28,31] (Figure 5).

In terms of the time it takes to carry out an evaluation using the screening tool, the shortest time needed to use STRONGkids could facilitate its integration into pediatric teams compared to other time-consuming tools. In addition, it could be of particular benefit in those point-of-care settings with a high number of patients attending or in low-resource settings. However, anthropometric assessment in screening tools could help to detect patients who are already malnourished upon admission, allowing measures to correct progressive nutritional deterioration to be established from admission. In this regard, the PYMS tool seems to show good results in its ability to integrate risk assessment and the nutritional status of the patient upon admission [31].

The STAMP tool was also included in five articles [28,36,39,41,43]. This tool showed worse diagnostic accuracy than PYMS in the comparison by Katsagoni et al. [36], as STAMP had a lower kappa value compared to the nutritionists, particularly when using HGC in the anthropometric assessment of PYMS (kPYMS_HGC = 0.48 vs. kSTAMP_WHO = 0.28). Similar results were found in the study by Gerasidimis et al. [19], where STAMP had a kappa value of 0.34 compared to nutritionists, whereas PYMS had a kappa value of 0.51. However, Marderfeld et al. [43] obtained better results with STAMP in their comparison with nutritionists (AUC 0.863). Both studies [36,43] use validation by a nutritionist, but it should be noted that the sample size was larger in the study by Katsagoni et al. [37], which included 907 patients, compared to that by Marderfeld et al. [43] that included only 60.

Another commonly used tool in the evaluated studies is the PYMS scale [28,36,37,39,44]. It showed good agreement with STAMP (kappa = 0.79) in the study by Bang et al. [39], but this association is weaker when comparing these two tools with STRONGkids for detecting malnutrition based on anthropometry in burn patients (STRONGkids vs. PYMS kappa = 0.39; STRONGkids vs. STAMP kappa = 0.37). This lack of agreement between STAMP and STRONGkids has also been reported in other systematic reviews [27], as well as moderate agreement between STAMP, STRONGkids, and PYMS [11] or between STAMP and PYMS [19].

Regarding the comparison between STRONGkids and PYMS, Beser et al. [37] observed a substantial variation in risk classification between these two tools, both upon admission and discharge of patients, with PYMS demonstrating greater sensitivity and specificity than STRONGkids across all age groups. Additionally, PYMS, unlike STRONGkids, recorded higher scores at admission in the group that experienced weight loss during hospitalization (*p* = 0.02), for weight-to-height ratio (*p* = 0.02), and for weight for age and BMI (*p* = 0.03) [37].

The good performance of the PYMS tool in assessing nutritional risk has been described in other studies [27,30,46], where it showed a better correlation with anthropometry compared to STAMP and STRONGkids [30]. STRONGkids yielded poorer results in our review compared to those published in other studies or systematic reviews [9,10,16,47], where it even showed a strong correlation with the state of acute malnutrition upon admission [10].

The PNSS scale was developed by Lu et al. [28] with the aim of creating a screening tool that considers clinical diagnoses in pediatric populations in China. Its validation was carried out by comparing it with PYMS, STAMP, STRONGkids, and anthropometric evaluation, as well as with a nutritional assessment in part of the sample. In this study, PYMS achieved the best concordance with the nutritionist’s evaluation (kappa = 0.5), with STAMP and PNSS showing intermediate results, and STRONGkids obtaining the lowest concordance (kappa = 0.3) [28].

Regarding hospital stay, the increase in its duration for those with higher nutritional risk observed in our review has already been widely observed in the literature [9,11,20,26,29].

Regarding pediatric oncology patients, SCAN was not able to adequately categorize malnourished patients, using anthropometric assessment as the reference [42]. NRS-PC was developed by Gallo et al. [40] and assesses weight loss, physical activity, dietary changes, stool patterns, and other gastrointestinal symptoms, in addition to BMI-based anthropometry [40]. NRS-PC achieved an AUC of 0.9 with a sensitivity of 98%, but when compared to SCAN, it is not really an adequate reference standard for the diagnosis of malnutrition [27]. However, NRS-PC achieved a significantly higher AUC (0.75; 95% CI: 0.67, 0.82) than SCAN (0.67; 95% CI: 0.58, 0.75) in detecting children with low muscle mass identified by bioelectrical impedance analysis [40].

In order to address the heterogeneity of the data in the studies analyzed, stratification by different variables was studied. First, regarding geography, the three more common screening tools—STAMP, STRONGkids and PYMS—were used in studies carried out in Europe [36,38,40,41,42,43] or in other countries, and anthropometric assessment was the most frequent gold standard employed, with assessment by a nutritionist being used as a reference independently of the country [36, 43]. Second, only three studies were carried out in patients with specific pathologies: burn patients [39] and oncology patients [40,42]; these last two studies are not comparable because although both evaluate the SCAN screening tool, only the study by Gallo et al. [40] used the bioelectrical impedance analysis to assess the degree of malnutrition. Finally, five of the eleven studies [28,36,37,38,44] included a sample of more than five hundred, with the PYMS nutritional screening tool being the most frequently evaluated tool and anthropometric assessment being the reference standard used in all of them with the exception of the study by Katsagoni et al. [36], although this assessment was performed in only 60.2% of the patients.

The results of our review are similar to those published in recent years in other systematic reviews [27,47], which conclude that STRONGkids and PYMS may be the most suitable tools for the early detection of patients at risk of malnutrition. Considering both, the PYMS tool had the highest correlation with assessment by a nutritionist. In addition, it allows for the detection of patients who will present weight loss during their admission, and it was the tool that obtained the best sensitivity and specificity in the studies analyzed.

The limitations of this review are marked by the considerable heterogeneity of the included studies, as noted in other previously published systematic reviews [16,27,47,48,49]. This makes it more challenging to compare the different tools and draw definitive conclusions. Such heterogeneity is evident in the included population, the patients’ pathologies, the nutritional tools used, and the reference standards employed and makes it difficult to perform a meta-analysis, which is another limitation of the study. One of the main challenges in making these comparisons is the lack of a universal definition for malnutrition or a gold standard that allows us to validate these tools [5,14,15,23,27,30,31,48]. However, nutritional assessment by a nutritionist or physician is usually considered to be the highest quality standard [25,31], so it is used as close to the gold standard in most studies when validating a screening tool [19,22,24,28].

These limitations highlight the need to develop a gold standard in pediatric nutritional screening. The ideal tool should be easy to apply (able to be used for different age ranges with simple parameters, and must be feasible for different health professionals) but also reliable (correlation with degree of malnutrition) and capable of detecting the risk of long-term malnutrition. For its development, a multicenter approach with different countries and health systems and the use of new technologies could be useful.

## 5. Conclusions

In our systematic review, we identified a variety of nutritional screening tools, some of which are newly developed. A high level of heterogeneity was observed among the selected studies, along with the absence of a gold standard that would allow for easy comparison between the different available tools. Despite these challenges, PYMS demonstrated good concordance with evaluations made by nutritionists and with anthropometric evaluations, and it may also serve as a useful predictor of weight deterioration during hospital admission. Therefore, it should be considered when selecting a nutritional screening tool.

## Figures and Tables

**Table 1:** PICOS criteria (Population, Intervention, Comparison, Outcomes, and Studies) for inclusion of studies.

Inclusion Criteria	
Population	Pediatric Population (0–18 Years)
Intervention	Use of nutritional screening tools to identify nutritional risk in pediatric population
Comparison	Comparison with another nutritional screening tool, anthropometric assessment, or nutritional evaluation conducted by qualified nutritionist
Outcome	Relationship between different nutritional screening tools, sensitivity, specificity, negative predictive value, and positive predictive value
Studies	Observational, cross-sectional, evaluation, and validation studies

**Table 2:** Results of QUADAS-2. ✔ = low risk of bias; ✖ = high risk of bias; 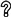 = uncertain risk.

Article	Applicability	Flow and Timeline	Reference Standard	Diagnostic Test to be Evaluated	Patient Selection
Maciel et al. (2020; Brazil) [35]	✔	✔	✖	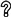	✔
Katsagoni et al. (2021; Greece) [36]	✔	✖	✔	✔	✔
Beser et al. (2018; Turkey) [37]	✔	✔	✖	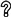	✔
De Longueville et al. (2018, Belgium) [38]	✔	✔	✖	✔	✔
Bang et al. (2018; Korea) [39]	✔	✔	✖	✔	✔
Gallo et al. (2021, Hungary) [40]	✔	✔	✖	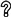	✔
Lu et al. (2018; China) [28]	✔	✖	✔	✔	✔
Pérez-Solís et al. (2020; Spain) [41]	✔	✔	✖	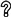	✔
Cañedo et al. (2022; Spain) [42]	✔	✔	✖	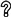	✔
Marderfeld et al. (2019; Israel) [43]	✔	✔	✔	✔	✔
Mckirdy et al. (2021; United Kingdom) [44]	✔	✖	✔	✔	✔

**Table 3:** Descriptive summaries of the selected studies.

Author, Year, and Country	Population (n; age; %female)	Screening Tool	Reference Standard Used	Main Findings
Maciel et al (2020; Brazil) [35]	Patients attending the Emergency Unit and under observation (n = 271; 1 month—10 years; 43.6% female)	Screening Tool for Risk on Nutritional Status and Growth (STRONGkids)	Anthropometric measurements (World Health Organization (WHO) standards), including mid-upper arm circumference	Weak correlation between nutritional screening tool and anthropometry
Katsagoni et al (2021; Greece) [36]	Hospitalized patients (n = 1506, 907 evaluated by a nutritionist; 1–16 years, median 5.7 years; 42% female)	Screening Tool for the Assessment of Malnutrition in Pediatric (STAMP); Pediatric Yorkhill Malnutrition Score (PYMS)	Validation by pediatric clinical nutritionist and anthropometric measurements (WHO standards and Hellenic Growth Charts (HGCs))	PYMS was superior compared to STAMP based on its sensitivity and specificity against global clinical criterion of nutritionists (kappa PYMS_HGC = 0.48 vs. kappa STAMP_WHO = 0.28)
Beser et al (2018; Turkey) [37]	Hospitalized patients (n = 1513; 1 month—18 years, mean 4.4 years; 43.6% female)	PYMS; STRONGkids	Anthropometric measurements (WHO standards)	Use of different tools resulted in substantial variation in classification of children’s malnutrition risk
De Longueville et al. (2018, Belgium) [38]	Hospitalized patients (n = 2657; 1 month—26.9 years, mean 4.8 years; 42.6% female)	EvalNut program; Pediatric Nutritional Risk Score (PNRS)	Anthropometric measurements (WHO standards)	Including nutrition program in medical record can be useful to spark interest among caregivers and is particularly valuable for nutrition team
Bang et al (2018; Korea) [39]	Hospitalized burn patients (n = 100; 3 months—16.5 years, mean 7.6 years; 48% female)	STRONGkids; PYMS; STAMP	Anthropometric measurements (WHO standards)	STRONGkids, PYMS, and STAMP tools are useful and practical for identifying patients with acute burn injuries at nutritional risk
Gallo et al (2021, Hungary) [40]	Oncology patients (n = 109; 3–21 years, mean 11.3 years; 41.2% female)	Screening Tool for Childhood Cancer (SCAN); NRS-PC (Nutrition Risk Screening for Pediatric Cancer)	Comparative validation between screening tools and bioelectrical impedance analysis	Patients with high Body Mass Index (BMI) would benefit from screening with NRS-PC; in cases of low BMI, bioimpedance measures provide more accurate information about muscle mass and nutritional risk (*p* < 0.001)
Lu et al (2018; China) [28]	Hospitalized patients (n = 2632, 847 evaluated by a nutritionist; 1 month—17 years, mean age 2.9 years; 52.2% female)	Pediatric Nutritional Screening Score (PNSS); PYMS; STAMP; STRONGkids	PYMS, STAMP, and STRONGkids; anthropometric measurements (WHO standards); assessment by nutritionist	PNSS is simple and reliable screening tool for malnutrition risk
Pérez-Solís et al (2020; Spain) [41]	Hospitalized patients (n = 81; 1 month—16 years, mean age 4.1 years; 43.2% female)	STAMP; STRONGkids	Anthropometric measurements (WHO standards)	STAMP and STRONGkids showed moderate agreement (Cohen’s kappa = 0.47) with high sensitivity but low specificity for diagnosing malnutrition
Cañedo et al (2022; Spain) [42]	Oncological patients (n = 49; age 0–18 years, mean age 9.4 years; 51% female)	SCAN	Anthropometric measurements (WHO and American Society for Parenteral and Enteral Nutrition standards), including mid-upper arm circumference (Abdel-Rahman et al. standards) [45]	Easy to use; discrepancy between SCAN classification and anthropometric classification (*p* = 0.91 for BMI and *p* = 0.11 for weight-to-height ratio)
Mardterfeld et al (2019; Israel) [43]	Hospitalized patients (n = 60; 1–17 years, mean age 7.2 years; 38.3% female)	STAMP	Assessment by nutritionist	STAMP is valid tool for malnutrition screening in hospitalized children; Area Under the Curve (AUC) was 0.86
Mckirdy et al (2021; United Kingdom) [44]	Hospitalized patients and children attending follow-up outpatient gastroenterology clinics (n = 595, 357 evaluated by a nutritionist; 5–16 years, mean age 10.4 years; 45% female)	PYMS; Handgrip Strength (HGS)	Anthropometric measurements (WHO-UK); PYMS; assessment by nutritionist	HGS predicts fat-free mass and could be used as complementary method to detect nutritional risk and need for further assessment and nutritional intervention upon hospital admission

AUC: Area Under the Curve; BMI: Body Mass Index; HGC: Hellenic Growth Charts; HGS: Handgrip Strength; NRS-PC: Nutrition Risk Screening for Pediatric Cancer; PNRS: Pediatric Nutritional Risk Score; PNSS: Pediatric Nutritional Screening Score; PYMS: Pediatric Yorkhill Malnutrition Score; SCAN: Screening Tool for Childhood Cancer; STAMP: Screening Tool for the Assessment of Malnutrition in Pediatrics; STRONGkids: Screening Tool for Risk on Nutritional Status and Growth; WHO: World Health Organization.

**Table 4:** Results of diagnostic accuracy of selected studies.

Reference	Screening Tool	NPV (95% CI) %	PPV (95% CI) %	Specificity (95% CI) %	Sensitivity (95% CI) %	AUC (95% CI) %	Kappa Value (95% CI)
Maciel et al. (2020; Brazil) [35]	STRONGkids	67.2 (54.9–79.5)	49.8 (43.0–56.6)	26.7 (19.5–33.9)	84.8 (78.4–91.2)	-	-
Katsagoni et al. (2021; Greece) [36]	PYMS	96.2	57.7	82.5	88.2	-	Kappa PYMS_HGC = 0.48 (0.43–0.53)
	STAMP	93.8	38.7	63.8	84.4	-	Kappa STAMP_WHO = 0.28 (0.23–0.33)
Beser et al. (2018; Turkey) [37]	STRONGkids (1 m–10 y; weight for age; Standard Deviation, SD)	-	-	90.3	70.3	-	-
	STRONGkids (5–18 y; BMI; SD)	-	-	94.8	72.3	-	-
	PYMS (1 m–10 y; weight for age; SD)	-	-	95.4	86.6	-	-
	PYMS (5–18 y; BMI; SD)	-	-	97.3	87.8		
Gallo et al. (2021, Hungary) [40]	SCAN	-	-	-	-	0.67 (0.58–0.75) (vs. Bioelectrical Impedance Analysis)	-
	NRS- PC	98 (91–100)	58 (47–68)	62 (51–71)	98 (90–100]	0.9 (vs. SCAN)	-
		-	-	60	75	0.75 (0.67–0.82) (vs. Bioelectrical Impedance Analysis)	-
Lu et al. (2018; China) [28]	PNSS	92 (89–94)	49 (44–54)	71 (67–74)	82 (76–87)	0.881 (vs. dietitian)	0.435 (0.373–0.498)
	PYMS	86.6	75.3	95.4	49	-	0.506 (0.431–0.581)
	STAMP	91	53.4	81.8	72	-	0.479 (0.403–0.555)
	STRONGkids	92.6	37.7	60.2	83.2	-	0.304 (0.239–0.369)
Pérez-Solís et al. (2020; Spain) [41]	STAMP	100 (88.3–100)	17.3 (9.4–29.7)	40.3 (29.7–51.8)	100 (70.1–100)	-	STAMP vs. STRONGkids k = 0.471
	STRONGkids	100 (90.1–100)	19.6 (10.7–33.2)	48.6 (37.4–59.9)	100 (70.1–100)	-	-
Marderfeld et al. (2019; Israel) [43]	STAMP	83.3	93.7	76.9 (49.74–91.82)	95.7 (85.75–98.83)	0.863 (0.72–1.00) (vs. dietitian)	-
Mckirdy et al. (2021; United Kingdom) [44]	HGS measurement; criterion value: z score = −0.81 (age adjusted)	96	16	56	79	0.72 (vs. dietitian)	-
	HGS measurement; criterion value: z score = −1.2 (height adjusted)	96	19	69	70	0.71 (vs. dietitian)	-

AUC: Area Under Curve; BMI: Body Max Index; Confidence Interval: IC; HGC: Hellenic Growth Charts; HGS: Handgrip Strength; NPV: Negative Predictive Value; NRS-PC: Nutrition Risk Screening for Pediatric Cancer; PNRS: Pediatric Nutritional Risk Score; PNSS: Pediatric Nutritional Screening Score; PPV: Positive Predictive Value; PYMS: Pediatric Yorkhill Malnutrition Score; SCAN: Screening Tool for Childhood Cancer; SD: Standard Deviation; STAMP: Screening Tool for the Assessment of Malnutrition in Pediatrics; STRONGkids: Screening Tool for Risk on Nutritional Status and Growth; WHO: World Health Organization.

## Supplementary Materials

The following supporting information can be downloaded at: https://www.mdpi.com/article/10.3390/nu17030433/s1, Table S1: PRISMA 2020 checklist of the reporting this systematic review; Table S2: PRISMA 2020 abstract checklist. Table S3: Full-text articles assessed for eligibility but not included in the review.
