# Supplementary material for: Nutritional Screening Tools in the Pediatric Population: A Systematic Review
Source: Nutrients. 2025 Jan 24;17(3):433. doi: 10.3390/nu17030433 (PMC11820693; doi:10.3390/nu17030433)
Supplement: Supplementary file 1 [file nutrients-17-00433-s001.zip › nutrients-3390947-supplementary.pdf]

Supplementary Materials

Table S1. PRISMA 2020 checklist of the reporting this systematic review.

Section and Topic	Item #	Checklist item	Location where item is reported
**TITLE**			
Title	1	Identify the report as a systematic review.	Lines 67-68
**ABSTRACT**			
Abstract	2	See the PRISMA 2020 for Abstracts checklist.	Table S2
**INTRODUCTION**			
Rationale	3	Describe the rationale for the review in the context of existing knowledge.	Lines 49-60
Objectives	4	Provide an explicit statement of the objective(s) or question(s) the review addresses.	Lines 61-65
**METHODS**			
Eligibility criteria	5	Specify the inclusion and exclusion criteria for the review and how studies were grouped for the syntheses.	Lines 71-73
Information sources	6	Specify all databases, registers, websites, organisations, reference lists and other sources searched or consulted to identify studies. Specify the date when each source was last searched or consulted.	Lines 75-80
Search strategy	7	Present the full search strategies for all databases, registers and websites, including any filters and limits used.	Lines 75-80
Selection process	8	Specify the methods used to decide whether a study met the inclusion criteria of the review, including how many reviewers screened each record and each report retrieved, whether they worked independently, and if applicable, details of automation tools used in the process.	Lines 94-102
Data collection process	9	Specify the methods used to collect data from reports, including how many reviewers collected data from each report, whether they worked independently, any processes for obtaining or confirming data from study investigators, and if applicable, details of automation tools used in the process.	Lines 89-100
Data items	10a	List and define all outcomes for which data were sought. Specify whether all results that were compatible with each outcome domain in each study were sought (e.g. for all measures, time points, analyses), and if not, the methods used to decide which results to collect.	Lines 71-80
	10b	List and define all other variables for which data were sought (e.g. participant and intervention characteristics, funding sources). Describe any assumptions made about any missing or unclear information.	Lines 71-80
Study risk of bias assessment	11	Specify the methods used to assess risk of bias in the included studies, including details of the tool(s) used, how many reviewers assessed each study and whether they worked independently, and if applicable, details of automation tools used in the process.	Lines 144-149
Effect measures	12	Specify for each outcome the effect measure(s) (e.g. risk ratio, mean difference) used in the synthesis or presentation of results.	Table 3

Section and Topic	Item #	Checklist item	Location where item is reported
Synthesis methods	13a	Describe the processes used to decide which studies were eligible for each synthesis (e.g. tabulating the study intervention characteristics and comparing against the planned groups for each synthesis (item #5)).	Lines 112-118
	13b	Describe any methods required to prepare the data for presentation or synthesis, such as handling of missing summary statistics, or data conversions.	Page 8
	13c	Describe any methods used to tabulate or visually display results of individual studies and syntheses.	Page 8
	13d	Describe any methods used to synthesize results and provide a rationale for the choice(s). If meta-analysis was performed, describe the model(s), method(s) to identify the presence and extent of statistical heterogeneity, and software package(s) used.	Line 168<br>Line 171
	13e	Describe any methods used to explore possible causes of heterogeneity among study results (e.g. subgroup analysis, meta-regression).	Lines 299-312
	13f	Describe any sensitivity analyses conducted to assess robustness of the synthesized results.	Not applicable
Reporting bias assessment	14	Describe any methods used to assess risk of bias due to missing results in a synthesis (arising from reporting biases).	Figure 2
Certainty assessment	15	Describe any methods used to assess certainty (or confidence) in the body of evidence for an outcome.	Figure 2
**RESULTS**			
Study selection	16a	Describe the results of the search and selection process, from the number of records identified in the search to the number of studies included in the review, ideally using a flow diagram.	Lines 112-118
	16b	Cite studies that might appear to meet the inclusion criteria, but which were excluded, and explain why they were excluded.	Table S3
Study characteristics	17	Cite each included study and present its characteristics.	Table 2
Risk of bias in studies	18	Present assessments of risk of bias for each included study.	Figure 2
Results of individual studies	19	For all outcomes, present, for each study: (a) summary statistics for each group (where appropriate) and (b) an effect estimate and its precision (e.g. confidence/credible interval), ideally using structured tables or plots.	Table 3
Results of syntheses	20a	For each synthesis, briefly summarise the characteristics and risk of bias among contributing studies.	Figure 2
	20b	Present results of all statistical syntheses conducted. If meta-analysis was done, present for each the summary estimate and its precision (e.g. confidence/credible interval) and measures of statistical heterogeneity. If comparing groups, describe the direction of the effect.	Lines 169-173
	20c	Present results of all investigations of possible causes of heterogeneity among study results.	Line 323-

Section and Topic	Item #	Checklist item	Location where item is reported
			330
	20d	Present results of all sensitivity analyses conducted to assess the robustness of the synthesized results.	Figure 2
Reporting biases	21	Present assessments of risk of bias due to missing results (arising from reporting biases) for each synthesis assessed.	Figure 2
Certainty of evidence	22	Present assessments of certainty (or confidence) in the body of evidence for each outcome assessed.	Table 3
**DISCUSSION**			
Discussion	23a	Provide a general interpretation of the results in the context of other evidence.	Line 216-318
	23b	Discuss any limitations of the evidence included in the review.	Lines 320-330
	23c	Discuss any limitations of the review processes used.	Lines 320-330
	23d	Discuss implications of the results for practice, policy, and future research.	Lines 331-336
**OTHER INFORMATION**			
Registration and protocol	24a	Provide registration information for the review, including register name and registration number, or state that the review was not registered.	Line 73-74
	24b	Indicate where the review protocol can be accessed, or state that a protocol was not prepared.	Line 73-74
	24c	Describe and explain any amendments to information provided at registration or in the protocol.	Not applicable
Support	25	Describe sources of financial or non-financial support for the review, and the role of the funders or sponsors in the review.	Line 351
Competing interests	26	Declare any competing interests of review authors.	Line 356
Availability of data, code and other materials	27	Report which of the following are publicly available and where they can be found: template data collection forms; data extracted from included studies; data used for all analyses; analytic code; any other materials used in the review.	Line 354-355

Table S2. PRISMA 2020 abstract checklist.

Section and Topic	Item #	Checklist item	Reported (Yes/No)
**TITLE**			
Title	1	Identify the report as a systematic review.	Yes
**BACKGROUND**			
Objectives	2	Provide an explicit statement of the main objective(s) or question(s) the review addresses.	Yes
**METHODS**			
Eligibility criteria	3	Specify the inclusion and exclusion criteria for the review.	Yes
Information sources	4	Specify the information sources (e.g. databases, registers) used to identify studies and the date when each was last searched.	Yes
Risk of bias	5	Specify the methods used to assess risk of bias in the included studies.	Yes
Synthesis of results	6	Specify the methods used to present and synthesise results.	Yes
**RESULTS**			
Included studies	7	Give the total number of included studies and participants and summarise relevant characteristics of studies.	Yes
Synthesis of results	8	Present results for main outcomes, preferably indicating the number of included studies and participants for each. If meta-analysis was done, report the summary estimate and confidence/credible interval. If comparing groups, indicate the direction of the effect (i.e. which group is favoured).	Yes
**DISCUSSION**			
Limitations of evidence	9	Provide a brief summary of the limitations of the evidence included in the review (e.g. study risk of bias, inconsistency and imprecision).	Yes
Interpretation	10	Provide a general interpretation of the results and important implications.	Yes
**OTHER**			
Funding	11	Specify the primary source of funding for the review.	Yes
Registration	12	Provide the register name and registration number.	Yes

Table S3. Full-text articles assessed for eligibility but not included in the review.

	Type Study	Aims/Intervention	Age	Reason of exclusion
**Phong et al (2020) (61)**	Retrospective observational study	To compare mid-upper arm circumference scores with anthropometric measurements (BMI-for-age and weight-for-height ratio) for the diagnosis of malnutrition in patients with Cystic Fibrosis and compare the degree of malnutrition with lung function	Infants-19 years	Not use a validated pediatric nutritional screening tool
**Tessema et al (2020) (62)**	Retrospective observational study	To compare arm circumference with weight-height ratio for the diagnosis of malnutrition and to define optimal cut-off points for arm circumference to detect severe acute malnutrition in children under 5 years of age	6 months-5 years	Not use a validated pediatric nutritional screening tool
**Phillips et al (2020) (63)**	Retrospective observational study	Provide nutritional care to all patients by reducing the time needed to recognize malnutrition through the implementation of an automated malnutrition detection system based on anthropometric measurements	>1 month	Not use a validated pediatric nutritional screening tool<br>Include non-pediatric patients and do not provide data on the median or age range of the patients included
**Kumar et al (2020) (64)**	Retrospective observational study	To predict mortality based on anthropometric and non-anthropometric criteria	1-6 months	Only anthropometric parameters and no validated nutritional screening tools
